# Stress responses in surgical trainees during simulation-based training courses in laparoscopy

**DOI:** 10.1186/s12909-024-05393-3

**Published:** 2024-04-12

**Authors:** Maria Suong Tjønnås, Sébastien Muller, Cecilie Våpenstad, Johannes Tjønnås, Solveig Osborg Ose, Anita Das, Mariann Sandsund

**Affiliations:** 1 Department of Neuromedicine and Movement Science (INB), Faculty of Medicine and Health Sciences, NTNU, Norwegian University of Science and Technology, Trondheim, N-7491 Norway; 2 Department of Health Research, SINTEF Digital, SINTEF, P.O. Box 4760, Torgarden, Trondheim, NO-7465 Norway; 3 Department of Mathematics and Cybernetics, SINTEF Digital, SINTEF, P.O. Box 4760, Torgarden, Trondheim, NO- 7465 Norway; 4 Department of Clinical and Molecular Medicine (IKOM), Faculty of Medicine and Health Sciences, NTNU, Norwegian University of Science and Technology, Trondheim, N-7491 Norway; 5 The National Research Centre for Minimally Invasive and Image-guided Diagnostics and Therapy (MiDT), St. Olavs Hospital, Trondheim University Hospital, P.O. Box 3250, Prinsesse Kristinas Gate 5, Torgarden, Trondheim, NO-7006 Norway

**Keywords:** Stress response, Simulation-based learning, Laparoscopic skills, Surgical trainee, Heart rate variability, Cortisol, STAI-6

## Abstract

**Background:**

Simulation-based training courses in laparoscopy have become a fundamental part of surgical training programs. Surgical skills in laparoscopy are challenging to master, and training in these skills induces stress responses in trainees. There is limited data on trainees’ stress levels, the stress responses related to training on different laparoscopic simulators, and how previous experiences influence trainees’ stress response during a course. This study investigates physiologic, endocrine and self-reported stress responses during simulation-based surgical skills training in a course setting.

**Methods:**

We conducted a prospective observational study of trainees attending basic laparoscopic skills training courses at a national training centre. During the three-day course, participants trained on different laparoscopic simulators: Two box-trainers (the D-box and P.O.P. trainer) and a virtual reality simulator (LAPMentor™). Participants’ stress responses were examined through heart rate variability (HRV), saliva cortisol, and the State Trait Anxiety Inventory-6 (STAI-6). The correlation between previous laparoscopic experiences and stress response measurements was explored.

**Results:**

Twenty-four surgical trainees were included in the study. Compared to resting conditions, stress measures were significantly higher during simulation-training activity (the D-box (SDNN = 58.5 ± 23.4; LF/HF-ratio = 4.58 ± 2.71; STAI-6 = 12.3 ± 3.9, *P* < 0.05), the P.O.P trainer (SDNN = 55.7 ± 7.4; RMSSD = 32.4 ± 17.1; STAI-6 = 12.1 ± 3.9, *P* < 0.05), and the LAPMentor™ (SDNN = 59.1 ± 18.5; RMSSD = 34.3 ± 19.7; LF/HF-ratio = 4.71 ± 2.64; STAI-6 = 9.9 ± 3.0, *P* < 0.05)). A significant difference in endocrine stress response was seen for the simulation-training activity on the D-box (saliva cortisol: 3.48 ± 1.92, *P* < 0.05), however, no significant differences were observed between the three simulators. A moderate correlation between surgical experience, and physiologic and endocrine stress response was observed (RMSSD: *r*=-0.31; SDNN: *r*=-0.42; SD2/SD1 ratio: *r* = 0.29; Saliva cortisol: *r* = 0.46; *P* < 0.05), and a negative moderate correlation to self-reported stress (*r*=-0.42, *P* < 0.05).

**Conclusion:**

Trainees have a significant higher stress response during simulation-training compared to resting conditions, with no difference in stress response between the simulators. Significantly higher cortisol levels were observed on the D-box, indicating that simulation tasks with time pressure stress participants the most. Trainees with more surgical experience are associated with higher physiologic stress measures, but lower self-reported stress scores, demonstrating that surgical experience influences trainees’ stress response during simulation-based skills training courses.

**Supplementary Information:**

The online version contains supplementary material available at 10.1186/s12909-024-05393-3.

## Introduction

Laparoscopic surgery is challenging to master as it requires complex manoeuvring of long, stiff surgical instruments. It demands advanced hand-eye coordination to perform accurate movements while the surgeon watches a monitor with a two-dimensional image of a three-dimensional space [1]. Simulation-based training courses offer surgical trainees essential laparoscopic skills training by allowing trainees repeated practice of laparoscopic techniques and procedures in an environment free from any risk to patients’ health [2, 3]. Box-trainers and virtual reality (VR) simulators are commonly used in laparoscopic skills training [4]. Basic laparoscopic skills can be learned and practised on a box-trainer. This simulator consists of a box to mimic the abdomen, a stationary camera and monitor, and ports for the instruments. Box-trainers can be combined with a laparoscopic tower composed of surgical equipment used in the operating room. This allows for the practice of spatial orientation in the abdomen, and advanced suturing skills with the use of animal organs or plastic organs models. VR simulators are used for training of both basic laparoscopic skills and procedure-based simulations. These box-trainers and VR simulators vary in terms of structural and functional fidelity, and in task alignment. As a result, mastering them requires different techniques and skills [2].

Mastering laparoscopic skills demands high levels of cognitive and psychomotor performance of the learner and is associated with high levels of stress response [5–7]. Previous research has demonstrated that high levels of stress can reduce laparoscopic skills performance, particularly for inexperienced trainees who are susceptible to the negative effects of stress [6, 8]. Conversely, studies show that moderate levels of stress facilitate better performance, such as faster time to complete a task [9]. However, it is challenging to determine what level of stress influences performance, as the activation of stress responses are highly individual and context dependent [5, 6].

The stress response has been defined as the physical, mental, or emotional response to perceived increase in demand for motor, cognitive, or other performances [10]. In surgical skills training, a stress response may follow a challenging training task which is cognitively appraised as stressful [6]. The sympathetic nervous system is triggered, and the activation of the sympathetic-adreno-medullar (SAM) axis leads to a rapid response in the cardiovascular system with increase of heart rates, blood pressure and respiratory rates. At the same time, the hypothalamic-pituitary-adrenal (HPA) axis is triggered and stimulates the secretion of the stress hormone cortisol into the bloodstream, resulting in an elevated cortisol level, which influences many physiological functions [11–13]. The complex nature of the stress mechanisms leading to a stress response, makes it difficult to measure directly [12]. Several proxy measurements are needed to quantify and interpret changes in the stress response of surgical trainees [14]. A recent systematic review found that both heart rate variability (HRV) analysis and State Trait Anxiety Inventory (STAI) questionnaire were preferred as stress response measuring tools in studies conducted in surgical environments [15]. HRV measures reflects variations between consecutive interbeat intervals (IBI). Both the sympathetic and parasympathetic branches of the autonomic nervous system are involved in this regulation. In stressful situations, when sympathetic nervous activity increases, resting HRV decreases [16]. Subjective measures of stress rely on participants self-reporting their perceived level of stress [17]. The STAI questionnaire is widely used as a validated self-reporting stress assessment tool [18]. The STAI-6, a shortened form of STAI is often used to assess stress associated with performing surgical tasks [15, 19]. A change in the HRV measures is an indication of activation of the SAM-axis, which is highly correlated to the physiologic stress response, while an elevated cortisol level is the body’s endocrine stress reaction, triggered through the HPA-axis. The STAI-6 is able to capture the subjective perceived stress. To capture and describe the different aspect of a person’s stress responses, a combination of physiological and psychological markers of stress markers, could ensure a more reliable assessment than using a single marker.

Stress responses have been the focus of much research in surgical environments over the past decade [15]. The impact of stress has been quantified and described in a large body of literature, contributing to a better understanding of the causes and consequences [20, 21]. The majority of this research has focused on stress in clinical, experimental or interventional settings, where the stress response has been induced intentionally through high-stress tasks, or as exposure to high-stress environments [22, 23]. The level of stress response during educational skills training courses is less explored. Little is known about trainees’ natural stress responses in training course settings, stress responses related to training on different simulator modalities, and how previous laparoscopic experiences influence on trainees’ stress response during a course or training activity. The goal of simulation-based skills training courses is to help trainees develop a high-level of proficiency in technical skills. Knowledge of trainees’ stress responses during simulation-based training courses involving use of different modalities, could help us to identify barriers to effective learning outcomes.

The aim of this study was therefore to examine trainees’ stress response when training basic laparoscopic skills in a simulation-based training course setting. We measured trainees’ stress responses during training on three different simulators, investigating whether stress responses would differ between three simulation-training tasks. Stress responses was examined through a combination of physiological and endocrine measurements of stress response using HRV variables and saliva cortisol, and self-reporting of perceived stress using the STAI-6.

We hypothesised that surgical trainees would have significantly increased stress response activation when training on simulated laparoscopic tasks compared to resting conditions. We further hypothesised that there would be a difference between the three simulation-training tasks, and a negative correlation between previous experience and the level of stress response.

## Methods

### General

**Fig. 1:**
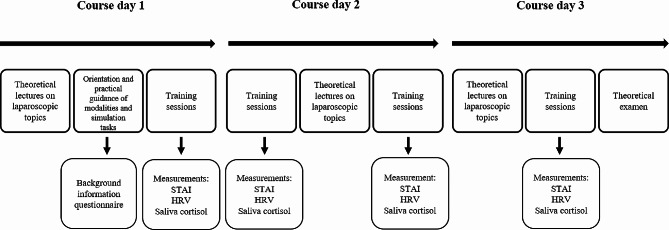
Schematic overview of the study design, measurements and the simulation-based training course timeline. HRV = heart rate variability, and STAI = State-Trait Anxiety Inventory scores

This was a prospective observational cohort study. Heart rate variability variables were used as primary indicators of stress, supported by an endocrine marker of stress, the saliva cortisol, and participants’ self-reported stress scores, the STAI-6 questionnaire. Data was collected at six courses from September 2019 through June 2022. The study is reported according to the STROBE Statement reporting guidelines with extensions for simulation-based research (Additional file 1) [24]. Ethical approval was obtained through the Regional Committees for Medical and Health Research Ethics (REC). Figure 1 shows a schematic overview of the study design, measurements, and simulation-based training course timeline.

### Participants and recruitment

Participants were recruited at the national centre for training of advanced laparoscopic surgery in Norway, which provides specialised educational courses for surgical trainees. The study was open to all surgical trainees with little or no previous experience in using laparoscopic techniques who had enrolled in mandatory courses in basic laparoscopic techniques, as a part of their surgical specialisation training. The exclusion criteria included medical conditions that affect or influence heart rate variability or hormone levels, i.e., arrhythmia or ongoing pregnancy. Trainees who had signed up for the courses, were invited to participate in the study by email. In addition, an oral invitation to join the study was given at the beginning of the courses. Written consent was obtained from all participants prior to joining the study. Participant demographic characteristics and work experience were collected through a questionnaire (Additional file 2).

### Simulators used for training laparoscopic skills

The three simulators used for the training of basic laparoscopic skills were two box-trainers, the D-box (Covidien Surgical Box, Mansfield, MA, USA) and the P.O.P. trainer (Optimist, Innsbruck, Austria), and a VR simulator, the LAPMentor™ (3D Systems, Littleton, CO, USA). Detailed descriptions of the simulators, simulation tasks and task requirements are reported in accordance with the Cheng et al. [24]. *Reporting standard for simulation studies* in Supplementary Table 1 (Additional file 3).

### The simulation-based training settings

The training courses lasted for three consecutive days. The simulation sessions were placed after or between theoretical lectures in laparoscopic surgery. The instructors gave the trainees orientation to each simulator, simulation tasks, and task requirements before the session started. The trainees were assigned to a simulator ahead of the sessions and trained for 1 h on each simulator before rotating to the next simulator. The simulation sessions lasted for 3 h on the first day, 5 h on the second day, and 3 h on the last day. On the D-box, and the LAPMentor™, the trainees operated alone, while the trainees were paired on the P.O.P. trainer. Guidance and feedback on performance and technique were given throughout all sessions by the instructors.

### Physiological stress markers

#### Heart rate variability (HRV)

HRV is the spontaneously occurring change in the time interval between successive heartbeats. This interval is highly correlated with the autonomic nervous system (ANS) and reflects the balance of sympathetic to parasympathetic contributions to cardiac rhythm modulation [25]. HRV analysis was based on examining IBI extracted from electrocardiogram (ECG) as time intervals between successive ECG R-waves [16]. HRV can be formulated using time-domain, frequency-domain, and nonlinear methods [26]. For this study, we focused on formulations indicating stress response commonly used for assessing physiological stress in surgical environments [15]:

#### The root mean square of successive differences of R-R intervals (RMSSD)

It reflects vagal tone and is highly correlated with high-frequency HRV. RMSSD is relatively free of respiratory influences. High RMSSD values indicate a strong respiratory sinus arrhythmia component, and high parasympathetic cardiac activation.

#### Standard deviation of the R-R intervals (SDNN)

It reflects all the cyclic components responsible for variability in the period of recording. SDNN was measured in milliseconds (ms), and NN here means “normal” beats, i.e., removing abnormal or false beats. The SDNN measures the autonomic influence on HRV. High SDNN values indicate a high parasympathetic cardiac activation. Longer recording periods of SDNN provide data about cardiac reactions to environmental stimulation. SDNN values are considered indicative of cardiovascular health, where low values are indicative of high cardiac risk [16, 25].

#### Low frequency (LF)/High frequency (HF)

In the frequency domain, HF components represents the parasympathetic activity, and LF components represents the activity associated with the sympathetic nervous system. The ratio LF/HF represents a balance of sympathetic-parasympathetic activity. A high LF/HF ratio indicates sympathetic dominance, while a low LF/HF ratio reflects greater parasympathetic activity relative to sympathetic activity. Lower HF power is associated with stress and anxiety and is linked to inhibition of the parasympathetic nervous system [25].

#### The Poincaré plot (SD2/SD1)

The Poincaré plot is used as a non-linear HRV analysis method. The plot is a graphical representation of the correlation between successive R-R intervals, where each R-R interval is plotted against the next interval. Poincaré plot analysis is a quantitative-visual technique where the geometry of the plot is essential in the interpretation of cardiologic function of the heart. The plot provides summary and detailed beat-to-beat information on the behaviour of the heart [27].

### Endocrine stress marker

#### Saliva cortisol

Cortisol is a biomarker of stress and can be measured in serum, saliva, hair and urine [28]. Saliva cortisol was used in the study. Saliva cortisol closely approximates the serum concentration, with a lag time of 2–3 min [13].

### Subjective measures of stress

#### State-trait anxiety inventory-short version (STAI-6)

This study used the STAI-6, a short version of the STAI questionnaire, with six items. Each item is a statement to which participants select their agreement on a four-point Likert scale (1 = not at all, 2 = somewhat, 3 = moderately 4 = very much). The total score results ranges from 6 to 24, where higher scores indicate greater stress response. The statements describe six different anxiety states, which can be related to cognitive, emotional, and physical response to stress. In a study on medical students performing simulated surgical tasks, baseline scores for STAI-6 were recorded to be 9.13 ± 3 [10]. The STAI-6 questionnaire form is included as a supplemental file (Additional file4).

### Data collection

All participants answered a questionnaire on their general health status, educational status, work experience, previous experience within simulation, laparoscopic simulation, laparoscopic operations, gaming, and fine motoric activities (Additional file 2). Heart rate recordings were collected using an ECG recorder (Actiwave Cardio, CamNtech, Cambridge, United Kingdom). The ECG recorder got attached to the participant’s chest upon arrival at the training facilities, and recordings of ECG signals in resting conditions were made before the participants started their training activity. In resting conditions, the participants first sat on a chair, with hands relaxed on a table, without talking, and repeated this procedure while standing. The resting condition lasted for a duration of 45–50 min [29]. The participant’s ECGs were recorded continuously throughout the simulation sessions. After the simulation sessions ended, the ECG recorder was removed, and the data was downloaded using a compatible software application (Actiwave Cardio analysis Software, CamNtech, Cambridge, UK). To exclude artifacts or non-simulation activities, and to allow for annotation of the simulation activities performed, the participants were video recorded using action cameras (GoPro HERO7, GoPro, Inc., San Mateo, US). Saliva cortisol was collected in resting condition and during the training activity with a cotton swab, Salivette Cortisol (Sarstedt, Numbrect, Germany). The samples were collected approximately 30 min into the resting and training sessions to avoid carry over effects [13]. Subjects were instructed to chew on the cotton swab for 2 min before returning it to the tube. Samples were kept at 5 °C and centrifuged at 1000 mph for 10 min to extract saliva and stored at -70 °C before analysis. The STAI-6 was rated at resting conditions and during the training activities when the participants were performing the main task of the simulation-training, i.e., knot tying.

### Data analysis

The ECG sampling rate was set at 256 Hz for the recordings. Data from the ECG recordings were post-processed using MATLAB® (MATLAB®, The MathWorks, Inc. Natick, US) and Python™ (Python™, Python Software Foundation, Fredericksburg, US) software. Ectopic beats, artifacts and data errors were removed from the ECG datasets using a HRV-analysis package [30]. The video recordings of the simulation-training activities were manually annotated for each participant and their simulation activities. The segments when participants were not performing simulation activities, were discarded from analysis. The first 30 min of each simulation-training activity were used for the HRV analysis.

A laboratory centre at the university hospital analysed the saliva samples. Analysis procedure of saliva cortisol included liquid extraction, then analysis with high pressure liquid chromatography. Deuterated cortisol was used as standard. Quantitation was performed using a 9-point standard curve with response ratio as a function of the quantity ratio between analyte and standard (Agilent 1290 high-pressure liquid chromatograph with Agilent 6465 Triple Quad LC/MS-MS detector).

The total STAI-6 scores were calculated by reversing the positive items (calm, relaxed, content) and summarized in accordance with Marteau and Bekker [31]. The total scores ranged between 6 and 24, where higher scores indicated a higher stress response. In the review article by Bekker et al. 2003, a “normal” score is considered approximately 10.2–10.8 [32].

### Statistical analysis

The sample size calculations were based on the HRV variables as the main outcome measures, and to achieve 88% power, samples of at least 21 participants were required to detect a large effect size [33]. The Shapiro‒Wilks test and Q-Q plot were used to assess the normality of the data. Data were expressed as mean difference, standard deviation for continuous variables, median and interquartile range (IQR) for age data, and numbers and percentages for categorical variables. Descriptive HRV data were presented as medians, quartiles, maximum and minimum values, and outliers. Repeated measures ANOVA was used to compare averages of HRV and cortisol measures between the resting condition and simulation-training activities. The P values were adjusted for multiple comparisons with Bonferroni correction. Significant effects were further analysed using a Tukey’s post hoc test. Pairwise comparisons with T-tests were used to reveal significant differences. The Mann-Whitney U test was used to compare subjective rated scores of the STAI-6 between resting conditions and simulation-training activities, and pairwise contrasts with T-tests were carried out to investigate whether there were any differences. The Spearman *r* correlation coefficient was used to measure the strength and direction of the correlation between participants’ characteristics, prior laparoscopic and simulation experience, gaming experience and fine motoric skills, and stress response measures recorded during the simulation-training activity. Data for the three simulation tasks were pooled and treated as one simulation-training activity in the correlation analysis. Positive and negative correlations were described on a scale from − 1 to + 1 [34]. Differences were considered significant at an alpha level of 0.05. All statistical procedures were carried out using IBM SPSS statistics 29 for Windows Software package.

## Results

### Participant characteristics and previous experience

For this study, 90 surgical trainees who had signed up for the mandatory course in basic laparoscopic technique were invited to participate by email. After screening in accordance with the inclusion/exclusion criteria, 26 participants were included in the study. Due to missing or corrupted data, two were excluded, leaving 24 participants in the final analysis. The participants were from different universities and regional hospitals across Norway. The median (IQR) age of the participants was 31.5 (5) years. Most of the participants (70.8%) had operative experience as main surgeon in the range of 0–49 times, while the rest (29.2%) had experience in the range of 50–199 times. 91.7% of the participants had previous experience with technical laparoscopic simulation in the range of 0–49 h, while one participant had 100 h, and one had 225 h of training experience. Table 1 shows the participants’ detailed characteristics, including experience as surgical trainees, experience with laparoscopic procedures and techniques, simulations, computer games and fine motoric activities.

**Table 1:** Participant characteristics, work experience as surgical trainees, previous experience with laparoscopic procedures and techniques, simulations, computer games and fine motoric activities (*n* = 24) [absolute and percent]

Participant characteristics (*n* = 24)		
	Number of participants	[%]
Gender		
Male	14	58.0
Female	10	42.0
Work experience as surgical trainee (months)		
0–12	16	66.7
12–24	5	20.8
24–36	3	12.5
Previous experience as main surgeon using laparoscopic procedures (number of occasions)		
0–9	6	25.0
10–49	11	45.8
50–99	5	20.8
100–199	1	4.2
Previous experience as assisting surgeon using laparoscopic procedures (number of occasions)		
0–9	1	4.2
10–49	15	62.5
50–99	3	12.5
100–199	5	20.8
Previous training in technical laparoscopic simulation (hours)		
None	3	12.5
1–9	12	50.0
10–49	7	29.2
100–199	1	4.2
200–249	1	4.2
Overall experience in playing computer games (years)		
None	15	62.5
10>	6	25.0
10<	3	12.5
Overall experience in fine motoric activities; handcraft, needlework, play of musical instruments, or other similar activities (years).		
None	12	50.0
10>	8	33.3
10<	4	16.7

% = percentage of

### Stress response measurements

**Fig. 2:**
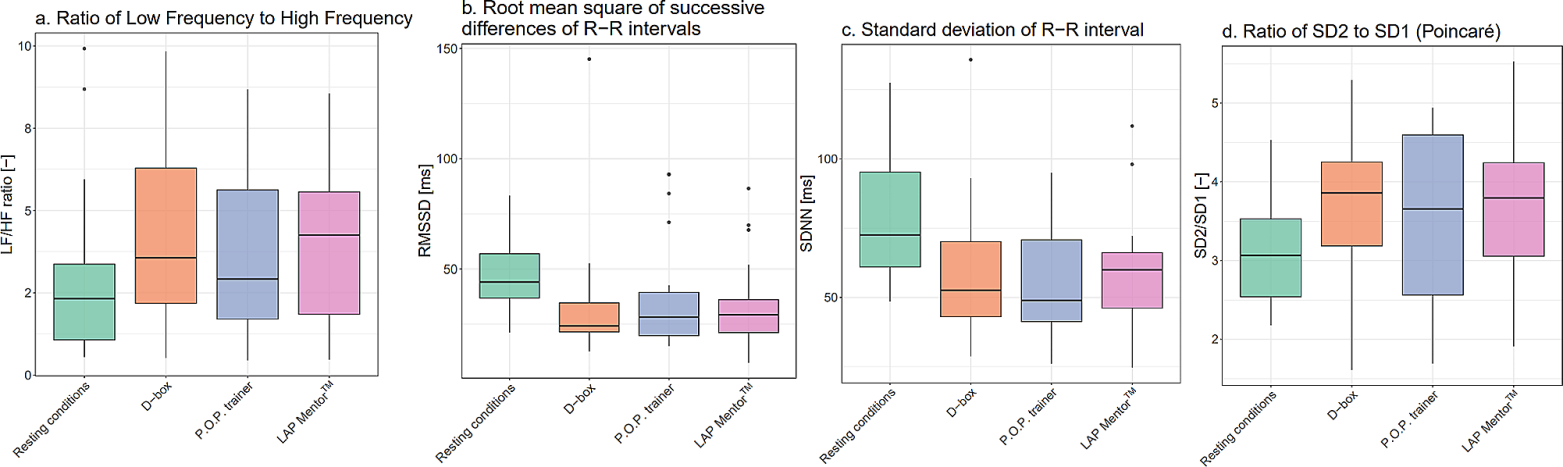
The boxplots of the median of LF/HF-ratio (**a**), RMSSD (**b**), SDNN (**c**) and SD2/SD1 ratio (**d**) data for 24 participants in resting conditions and during simulation-training activity on three simulators, the D-box, P.O.P. trainer and LAPMentor™. Data are presented as the median, minimum and maximum values, 25% and 75% quartiles, and **· =** outliers

Descriptive data for all measures of stress are presented in Table 2. The participants had a significant difference in SDNN between resting conditions and training activity on the D-box (*p* = 0.004), P.O.P. trainer (*p* < 0.001) and LAPMentor™ (*p* < 0.001). Similarly, there was a significant difference in RMSSD during training activity on the P.O.P. trainer (*p* < 0.001) and LAPMentor™ (*p* < 0.001) compared to resting conditions. However, there were no differences in RMSSD when training on the D-box compared to resting conditions. A significant increase was observed for the LF/HF ratio during the training activity on the D-box (*p* < 0.041) and the LAPMentor™ (*p* < 0.013) compared to the resting conditions, but no significant differences in stress response during training activity on the P.O.P. trainer. No difference was detected between the three simulation tasks based upon analysis of HRV variables. The analysis of the dispersion of the LF/HF ratio, RMSSD, SDNN and SD2/SD1 ratio data (Fig. 2) for resting conditions and the three simulation tasks showed that the data was similar with regard to the medians and quartiles.

### Saliva cortisol measurements

The differences in mean cortisol levels in the resting conditions compared to the three simulation tasks, revealed significant differences only during training activity on the D-box (*p* < 0.036).

### Self-reported stress

A significant difference in self-reported stress scores during training activity on the three simulators was observed. The participants scored higher stress scores when performing the training tasks compared to resting conditions. There was no difference in stress scores between the three simulation tasks.

**Table 2:** Descriptive statistics for all stress response measurements during simulation-training sessions

Stress measurements	Resting conditions	D-box	P.O.P. trainer	LAP Mentor™	Number of participants
HRV variables					
SDNN (ms)	77.3 ± 18.7	58.5 ± 23.4*	55.7 ± 17.4**	59.1 ± 18.5**	*N* = 22#
RMSSD (ms)	48.4 ± 15.7	33.8 ± 26.8	32.4 ± 17.1**	34.3 ± 19.7**	*N* = 22#
LF/HF-ratio	3.20 ± 2.40	4.58 ± 2.71*	4.38 ± 2.63	4.71 ± 2.64*	*N* = 23#
**Stress hormone activity**					
Saliva cortisol (nmol/L)	2.26 ± 1.14	3.48 ± 1.92*	3.40 ± 3.62	3.11 ± 2.71	*N* = 24
**Self-reported stress score**					
STAI-6 (score)	7.9 ± 2.1	12.3 ± 3.9*	12.1 ± 3.9*	9.9 ± 3.0*	*N* = 24

SD = standard deviation, N = number of participants, Saliva cortisol in nmol/L, STAI-6 = State-Trait Anxiety Inventory, the 6 item questionnaire, Mean = arithmetic mean, HRV variables: SDNN = standard deviation of all R-R intervals in ms, RMSSD = square root of the mean of the sum of the squares of differences between adjacent R-R intervals in ms, and LF/HF ratio, ratio of low-frequency (LF) to high-frequency power (HF), unitless

* Significant compared to resting conditions, *P* < 0.05

** Significant compared to resting conditions, *P* < 0.001

# Two/one participant(s) with missing data are excluded

### Correlation analysis of previous experience and stress measures

**Fig. 3:**
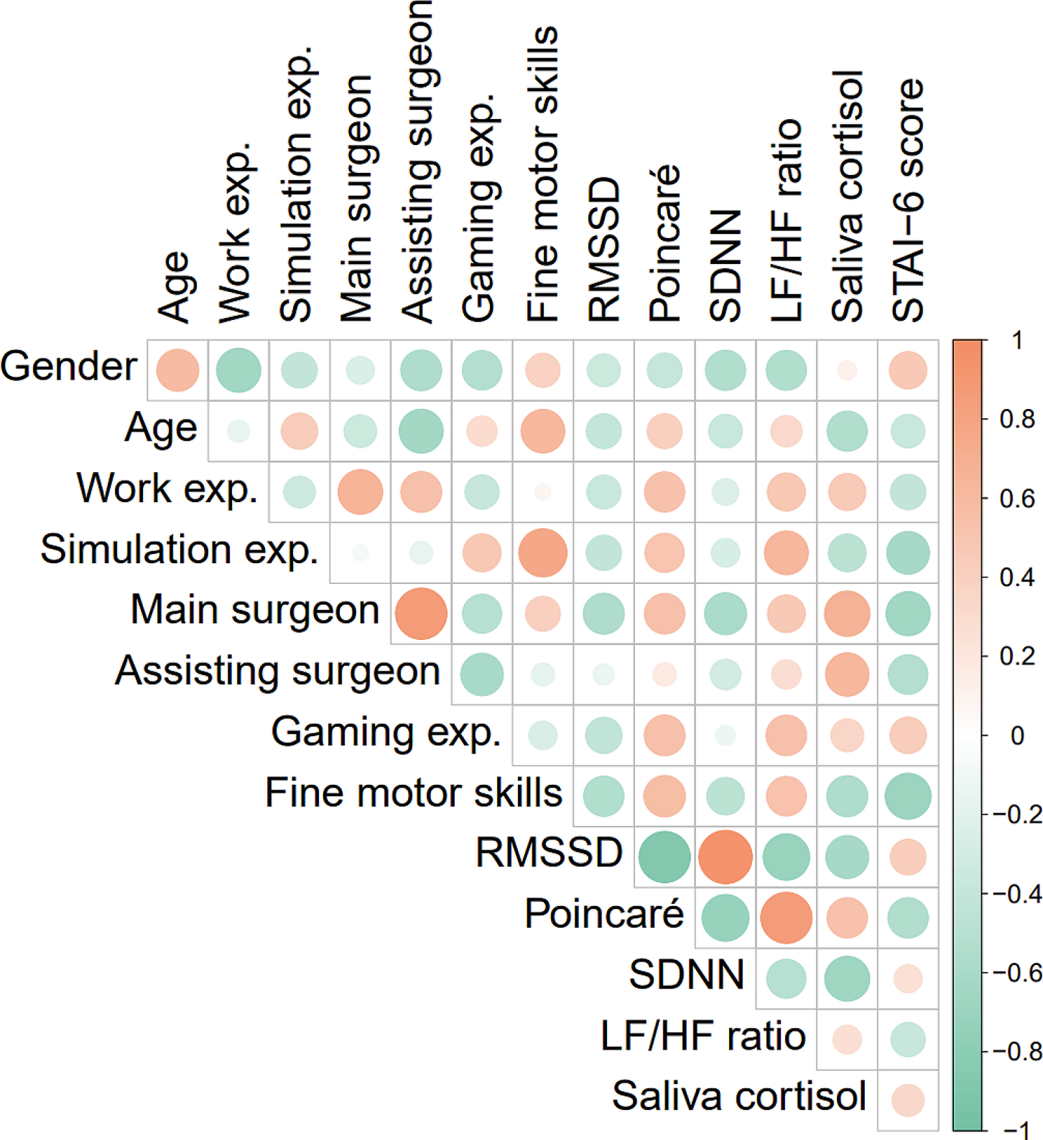
The correlation between participants’ characteristics; gender, age, work exp.= work experience as surgical trainees in months, simulation exp.= laparoscopic simulation experience, main surgeon = experience as main operative surgeon, assisting surgeon = experience as assisting surgeon, gaming exp.= computer game experience, fine motor skills = fine motoric activity, and stress markers; saliva cortisol = cortisol, STAI-6 score = State-Trait Anxiety Inventory scores, SDNN = standard deviation of all R-R intervals, RMSSD = root mean square of successive differences of R-R intervals, LF/HF ratio, ratio of low-frequency (LF) to high-frequency power (HF), and Poincaré = the Poincaré ratio, ratio of SD2 to SD1. Circle sizes and shade correspond to the strength of the correlation values. Red colour denotes positive values and green colour denotes negative values

Figure 3 shows the correlation matrix. The correlation coefficients between the variables are presented at the intersection of the corresponding rows and columns of the matrix. The numeric scale on the right of the matrix presents the strength of the correlation in terms of colour (red colour denotes positive values and green colour denotes negative values) and shade. The circle sizes and shade correspond to the strength of the correlation values. Correlation analysis (Fig. 3) showed a moderate association between participants’ experience as main surgeon and decreasing levels of RMSSD and SDNN (RMSSD: *r*=-0.31; SDNN: *r*=-0.42; *P* < 0.05) and increasing ratio values of SD2/SD1 (*r* = 0.29; *P* < 0.05), and levels of cortisol (*r* = 0.46; *P* < 0.05). Subjectively reported stress, was negatively correlated with trainees’ experience as the main operative surgeon (*r*=-0.42; *P* < 0.05).

Figure 3. Pearson’s *r* correlation coefficients between participants’ characteristics, their previous laparoscopic and simulation experience, gaming experience, fine motoric activity, HRV variables and saliva cortisol measures, and STAI-6 scores.

Figure 3. The correlation between participants’ characteristics; gender, age, work exp.= work experience as surgical trainees in months, simulation exp.= laparoscopic simulation experience, main surgeon = experience as main operative surgeon, assisting surgeon = experience as assisting surgeon, gaming exp.= computer game experience, fine motor skills = fine motoric activity, and stress markers; saliva cortisol = cortisol, STAI-6 score = State-Trait Anxiety Inventory scores, SDNN = standard deviation of all R-R intervals, RMSSD = root mean square of successive differences of R-R intervals, LF/HF ratio, ratio of low-frequency (LF) to high-frequency power (HF), and Poincaré= the Poincaré ratio, ratio between SD2 and SD1. Circle sizes and shade correspond to the strength of the correlation values. Red colour denotes positive values and green colour denotes negative values.

## Discussion

In this study, we examined the stress responses of surgical trainees during simulation-based training courses in basic laparoscopic skills. Trainees had a significantly higher stress response during simulation-training activity on all three simulator tasks compared to resting conditions. The HRV variables RMSSD and SDNN both showed decreased values, while the LF/HF ratio showed increased values, all indicating that the trainees experienced increased stress levels during the simulation-training activity. This suggests an activation of the physiological stress response, where the sympathetic nervous system is triggered with an immediate effect on the cardiovascular system with modulation of the heartbeat rhythms [6, 11]. As most of the participants had little or no previous experience in surgical skill simulation prior to the course, and were at the start of their training, the simulation task requirements might have been challenging to accomplish, causing the trainees to appraise the simulation tasks as threatening and consequently eliciting stress responses [12, 35]. In a study by Grantcharov et al. [36]. investigating acute stress and laparoscopic performance, demonstrated that the physiologic response to an event that is perceived as stressful was a decrease in SDNN and RMSSD values. Similar results have been described for studies in other simulation settings [23, 37]. Studies have shown that arousal of the stress system is beneficial in performing surgical procedures as this will help trainees focus their attention and motivation towards accomplishing the task requirements [37–39].

The self-reported stress scores were significantly higher for all three simulation tasks compared to resting conditions, demonstrating that the simulation-training activities in a course setting stimulate psychological stress. This confirms that the observed increases in physiologic stress response, as measured by HRV variables, were consistent with trainees’ subjective perception of stress. The results are comparable to previous research by Arora et al. [6] and Jones et al. [23] who used self-reported stress scores in combination with physiologic measures in simulation studies and reported positive correlations between the two measurement methods. Although the self-reported stress scores were directly related to training on the simulation tasks, there is an aspect of socio-evaluative factor involved when training in a course setting. In a training space where colleagues and peers are placed next to each other, there might be a risk for social-evaluative pressure in the environment influencing stress scoring [40]. Stress can be interpreted as a weakness, and admitting to having high levels of stress during a simulation task might leave trainees vulnerable to judgment by other course participants. To avoid the risk of stigmatization, trainees might have scored the STAI-6 too low. Jin et al. [41] described how the social pressures of surgical culture might negatively impact the execution of the tasks at hand. Awareness of the socio-evaluative factors affecting subjective measures of stress is an important factor to be considered in future studies.

During the course, three simulator modalities were used for training laparoscopic skills, providing three different types of simulation tasks regarding structural and functional fidelity, and task requirements. However, no difference in stress response levels was detected between the three simulation tasks based upon analysis of the HRV variables, which showed that all three simulation tasks were similar regarding the data dispersion, median and quartiles (Fig. 2). We suggest that this is because the task difficulty was similar for the three simulation tasks, which was reflected in similar HRV data. In a training course for basic skills, the task difficulty is calibrated for trainees who have little or no previous experience in laparoscopic skills or simulation, as well as for those with more experience but not yet on mastery level [2]. With similar task difficulty, the stress response activation will likely be on the same level, and a difference in stress response was therefore not detectable. These findings are in contrast to previous studies comparing different simulation-based laparoscopic tasks. VR simulation tasks have previously been demonstrated to induce stress in trainees and surgeons, while simulation tasks with time pressures performed on box-trainers, have similarly induced significant stress response in trainees [42–44]. The STAI-6 scores reflected the HRV variables, indicating that trainees did not perceive any difference between the three simulator tasks. This contrasts with previous research results by Tjønnås et al. [40] where surgical trainees were interviewed after training on box-trainers and VR simulators. Here it was found that trainees felt more distressed when training on the D-box simulator. The trainees reported that the task was particularly stressful because of the time pressure and socio-evaluative factors involved.

A significantly higher cortisol level was observed only for simulation-training activity on the D-box compared to resting conditions, indicating that this simulation-training task of the three simulation tasks performed during the course, seemed to elicit the highest stress response. To pass this simulation task, the participants had to complete the task within 1.5 min. The simulation task was designed to test bimanual-dexterity, hand-eye coordination, economy of motion and speed [4, 45]. This requires both cognitive and motor skills training [7]. For participants with little or no previous experience in training on this simulator, all these elements of laparoscopic skills training would likely have been difficult to master at this stage of their training, consequently eliciting a stress response [46]. To master new surgical skills, neurological pathways need to be established and new movement patterns have to be learned, which requires large cognitive resources of the trainees. Cognitive resources such as attention, focus and memory formation are needed in motor learning [47, 48]. Furthermore, the responsiveness of saliva cortisol is slower than that of HRV variables and STAI-6. The detection of elevated cortisol levels after a stressful event, has been demonstrated to take between 5 and 20 min [13]. Cortisol is rapidly degraded, and a sustained and large stress response is therefore needed to observe a sizable difference in cortisol levels [49]. Our study shows that the stress response was larger and sustained over a longer period when the trainees were training on the D-box, likely resulting from the additive effect of time pressure and technical demands, yielding a significant difference in saliva cortisol levels. Several studies have demonstrated how time pressure in simulated surgical skills training have additive effects, resulting in a larger stress response [6, 50].

Correlation analysis revealed that despite elevated physiologic stress levels during the courses, trainees with more surgical experience were associated with lower self-reported stress scores. This suggests that although there were increased stress levels recorded during the course, these stress levels were not perceived to be excessive by experienced trainees. With more surgical experience, the trainees might have developed coping skills in performing surgical procedures, and therefore are more tolerant to increased stress responses, and thus perceive demanding surgical tasks as less stressful compared to the ones less experienced [5, 51]. Previous studies have demonstrated that expert surgeons cope with higher level of stress than novice surgeons when performing the same procedures, which could be explained by the combination of acquired coping skills and clinical experience [9, 52]. A core premise for successful simulation-based training, is the ability to understand the functional alignment between the simulation task and real-life clinical task [53]. The advantage of having more surgical experience is that it can help trainees to relate the simulation task at hand to real-life clinical context [50, 54]. This understanding could have prompted the experienced trainees to be more engaged in accomplishing the task requirements during the course, and thus induce stress responses. Furthermore, being in a training setting where trainees are subject to evaluation of their technical skills by peers and senior surgeon consultants, it is likely that the experienced trainee had some level of social-evaluative pressures which elicited stress responses. Research by Flinn et al. [22] showed how socio-evaluative pressure by surgical instructors can elicit physiologic stress responses in participants during learning of surgical skills.

### Implications

The goal of simulation-based skills training courses is to help trainees develop a high-level of proficiency in technical skills, and thereby ensure patient safety. More knowledge of trainees’ stress levels in surgical simulation-based training courses could make an important contribution by identifying barriers to effective learning outcomes in achieving that goal. As stress has been demonstrated to influence trainees’ performance and training in interventional and experimental settings, it is likely that stress influences learning outcomes in simulation-based training course settings. In the present study, the trainees had increased saliva cortisol levels during training on the D-box. This simulation task, although low in structural fidelity, however, is considered to have high functional fidelity [55]. Combined with time pressure, the D-box seemed to induce high and sustained stress responses among trainees, providing evidence that time dependent simulation-tasks stimulate trainees to activate their stress systems. A heightened stress response, as measured by cortisol, has been demonstrated to be beneficial in forming long-term memory, an essential factor in learning [56, 57]. This is consistent with previous research on learning and stress [58]. Course facilitators should be aware of the effects of stress responses when implementing time dependent simulation tasks in course programs.

Furthermore, the findings could help inform trainees attending similar courses, to use this information to either try to decrease their physiological activation by employing stress management strategies [8, 59], or to accept the heightened stress activation and harness it to improve their performance and training during the courses [60].

In this study a correlation between previous surgical experience and stress levels was observed. This suggests that previous experience can influence the stress levels during simulation training activities, which can be beneficial for task engagement and learning outcomes in simulation-based training courses. However, with reduced working hours and less clinical exposure to laparoscopic procedures, gaining more surgical experience may not be easily achieved for surgical trainees [61]. To ensure adequate laparoscopic skills training, trainees could be encouraged to maximise their training by extended use of simulators. Self-directed simulator training at their own workplace could provide a solution to compensate for reduced surgical experience [62].

### Limitations

Of the 90 eligible surgical trainees that were invited to join the study, 26 trainees chose to participate. The trainees participated voluntarily in the study, so there was a possible risk of self-selecting bias. The main author, who recruited all the study participants, did not know any of the participants or have any background information about them when they were asked to join the study. A few of the participants had extensive training on box-trainers at their local hospital. With the advantage of having this training experience, these trainees might have presented with lower stress response. However, none of the participants reported having had their HRV parameters, cortisol levels and STAI-6 scores assessed prior to attending the courses. Without this knowledge, the potential for introducing bias by self-selection was therefore limited.

Stress responses are affected by many factors such as previous training experience on simulators [42]. The study design did not allow for pre-training sessions to control the baseline level of training experience of the participants, which means that participants may have had different baseline training experience when entering the study. This was an observational study where participants’ previous training experience was collected through self-reported questionnaire. This provided limited information on participants’ laparoscopic training and simulation experience.

The HRV variables and STAI-6 scores are extensively used as stress assessment tools in surgery. However, there are limitations regarding the ability to capture small differences or nuances in stress levels when using these methods. It has been suggested that HRV variables are not optimal in capturing smaller changes, as the raw ECG recordings go through extensive post processing, which may remove the small changes in the data, i.e. spikes in heart rate or ectopic beats [26]. As the STAI-6 is scored according to a 4-point Likert scale and comprises six questions, the questions could have been too broad to catch nuances or small differences in perceived stress. Matthews et al. (1999) described specific limitations of subjective stress assessment measures, suggesting that subjective stress measures may not be suitable for assessing stress changes during short periods of time, but rather more suitable for capturing long term changes in stress [63]. Considering these limitations, we based our data processing upon established guidelines and methodological articles on HRV data processing and supplemented with deeper analysis of the HRV data, and controlled for the subjective stress scores by asking the trainees to orally validate the scores post training sessions.

Saliva cortisol has a relatively slow response time to stress activation compared to other cortisol variables [13]. Using other endocrine stress markers might have provided a more accurate measure of acute stress response, i.e., serum cortisol; however, we had to balance the practicality of obtaining cortisol samples against the risk of disturbing trainees’ training flow, which could have affected the cortisol levels. We decided that collecting saliva cortisol was the least intrusive method of obtaining an endocrine stress marker.

The stress response is a result of many factors, and in this study, we mainly examined quantitative measures of the stress response. Other non-quantitative measures could also explain our results, such as socio-psychological factors, which could have influenced the results; however, we have chosen to focus on the biophysical stress response, and the analysis of psychological factors is considered out of scope for this study. This has been explored in a recent study by Tjønnås et al. [40].

#### Generalizability

The present study suggests that simulation-based training courses in laparoscopic skills induce significant stress in trainees. The results are transferable to future courses and to real-life clinical settings where trainees find themselves in situations where they are to perform technical procedures they have not yet mastered. The trainees will recognize that they will experience an elevated stress response and that previous surgical experience might moderate their stress experience.

## Conclusion

In this prospective observational study, we have shown significantly increased levels of stress using combined measures of HRV variables, saliva cortisol and self-reported assessment of stress (STAI-6) during simulation-based training activities on laparoscopic box-trainers and VR-simulators. Our results establish the presence of elevated stress levels in surgical trainees during educational course settings and identify time-dependent simulation tasks with high technical demands as particularly stressful. Correlation analysis reveals that the trainees with more surgical experience are associated with higher physiologic stress measures, but lower self-reported stress scores, demonstrating that surgical experience does not necessarily decrease physiologic stress measures but decreases perceived stress. These results confirm that the stress response is an important factor to consider in simulation-based training of surgical skills, and it warrants more research studies to investigate the effects of elevated stress levels on learning and performance outcomes in surgical skills training courses.

### Electronic supplementary material

Below is the link to the electronic supplementary material.

Supplementary Material 1

Supplementary Material 2

Supplementary Material 3

Supplementary Material 4

## Data Availability

The author confirms that all data generated or analysed during this study are included in this published article.

## Abbreviations

ANOVA: Analysis of variance
ANS: Autonomic nervous system
ECG: Electrocardiogram
HPA: Hypothalamic-pituitary-adrenal
HRV: Heart rate variability
Hz: Hertz
IBI: Interbeat-intervals
IQR: Interquartile range
LF/HF ratio: Ratio of Low Frequency (LF) to High Frequency (HF) power
Mph: Miles per hour
ms: Milliseconds
NN: Normal beats
REC: Regional committees for medical and health research ethics
RMSSD: Root mean square of successive differences of R-R intervals
R-R interval: The time elapsed between two successive R waves of the QRS signal on the electrocardiogram
R-waves: Representing ventricular depolarisation
SAM: Sympathetic-adreno-medullar
SD: Standard deviation
SDNN: Standard deviation of the R-R intervals
SD2/SD1: Ratio of SD2 to SD1
STAI-6: State Trait Anxiety Inventory-6 short version
STROBE: Strengthening the reporting of observational studies in epidemiology
VR: Virtual reality
