# Supplementary material for: Stress responses in surgical trainees during simulation-based training courses in laparoscopy
Source: BMC Med Educ. 2024 Apr 12;24:407. doi: 10.1186/s12909-024-05393-3 (PMC11010405; doi:10.1186/s12909-024-05393-3)
Supplement: Supplementary file 3 — Supplementary Material 3 [file 12909_2024_5393_MOESM3_ESM.docx]

**Additional file 3.**

Supplementary Table 1. Description of the simulator type, orientation to simulators and environment, simulation-based training, and feedback/debriefing. A modified version from the study by Tjønnås et al. (2022)(40).

| **Simulator type** | | | |
| --- | --- | --- | --- |
| Simulator make and model* | Box-trainer (D-box, Covidien Surgical Box, Mansfield, MA, USA). | Box-trainer (Pulsatile organ perfusion (P.O.P) box-trainer, Optimist, Innsbruck, Austria). | VR- simulator (LapMentor™, 3D systems, Littleton, USA). |
| Simulator functionality* | The D-box, a box-trainer had a fixed video camera and a light source inside the box, and entry holes for the laparoscopic instruments. Laparoscopic graspers were used for the simulation tasks. The box-trainer offered training of bi-manual dexterity, interpretation of 3D images on a 2D screen, depth perception, and eye-hand coordination. Limitations: the light source was fixed in one angle which could impact the visibility. | The P.O.P -trainer, a box-trainer which provided training on artificial organ models and real tissue using authentic laparoscopic instruments. The simulator had a top lid with entry ports for trocars. Both artificial organ models and porcine tissue were used to facilitate the simulation tasks. Limitations: the pulsating function which simulates circulation of liquids in real tissue was not available. | The LapMentor™, a VR-simulator provided training in a user interface with 3D data-generated images that offered a visually real-life experience. The simulator had built-in haptic technology. The module for basic training was set for exercises with pegs, appendectomy, and cholecystectomy. Limitations: the VR-simulator software would only accept a specific way of performing a procedure. |
| **Orientation to simulators and environment** | | | |
| Participants orientation to the simulator ahead of the training sessions* | Trainees were given outline of the simulation training task and the requirements, and practical guide in using the simulator technology. | Trainees were given outline of the simulation training task and the requirements, and practical guide in using the simulator technology, and instructions on how to work in pairs. | Trainees were given outline of the simulation training task and the requirements, and practical guide in using the simulator technology, which included handling the software and hardware. |
| Participant orientation to the environment ahead of the training session* | Trainees were assigned to one of six simulators, which were placed on bench tops, adjusted to trainees’ heights. Instructors were available on site, and trainees were allowed to engage in conversations or observations during training. | Trainees were assigned to one of five simulators, which were placed on bench tops with the associated computer screen and laparoscopic equipment mounted on a rack. Instructors were available on site, and trainees were allowed to engage in conversations or observations during training. | Trainees were assigned to one of two VR simulators. The VR simulator system was a self-contained unit with laparoscopic instruments and computer screens. Instructors were next to the trainees during training. Trainees were allowed to engage in conversations or observations during training. |
| Group vs. individual practice* | The trainees performed the simulation tasks alone. | The trainees performed the simulation task in pairs. | The trainees performed the simulation tasks alone. |
| **The simulation-based skills training** | | | |
| The simulation-training tasks | Pre-simulation training: Consisted of exercises to familiarize the trainee to the graspers, to using both hands, and the depth perception.  Main simulation-training task: Passing a thread through multiple loops in a pre-set path. | Pre-simulation training: Consisted of exercises to familiarize the trainee to the hand-eye coordination and depth perception, and suture exercises on artificial model organs.  Main simulation-training task: Suture and knot-tying exercises on porcine tissue. | Pre-simulation training: Consisted of exercises to familiarize the trainee to the VR interface, i.e., peg transfers.  Main simulation-training task: Full cholecystectomy and appendectomy procedures. |
| Learning objectives* | (1) Be familiarized with the laparoscopic instruments, practicing of bi-manual dexterity, training of hand-eye coordination, and to be able to interpret depth on the PC screen.  (2) Pass a thread through multiple loops in a pre-set path. | (1) Be familiarized with the laparoscopic instruments, and to be able to interpret depth on the PC screen.  (2) Practicing suture and knot-tying techniques.  (3) Accomplishing a surgical square knot. | (1) Be familiarized with the software and hardware of the simulator.  (2) Accomplishing a peg transfer simulation task.  (3) Accomplishing an appendectomy and a cholecystectomy. |
| Requirements/assessment* | Accomplishing the main simulation-training task within 1.5 minutes. | Accomplishing the main simulation-training task within 2.5 minutes. | Assessment of errors, time to complete task and other assessment metrics were logged in software. |
| Frequency/repetitions* | No restrictions on number of repetitions. | No restrictions on number of repetitions. | No restrictions on number of repetitions. |
| **Feedback/debriefing** | | | |
| Feedback* | Instructors gave feedback on the technical performance and provided guidance on performance if needed. | Instructors gave feedback on technical performance and provided guidance on performance if needed. | Instructors gave feedback on technical performance and provided guidance on performance. Computer program provided text-based feedback on errors, time to complete task, and other metrics. |
| Instructor presence* | One instructor was present throughout all simulation sessions. | Two to three instructors were present throughout all simulation sessions. | One instructor was present throughout all simulation sessions. |
| Instructor characteristics* | Instructors were medical educators with more than 10 years of experience. The main instructor held a PhD in medical technology with specialization in laparoscopic simulation. | Instructors were medical educators with more than 10 years of experience. Instructors were general surgeons with specialization in gastroenterology and gynecology, with 25-30 years of laparoscopic experience. | Instructors were medical educators with more than 10 years of experience. Instructors were general surgeons with specialization in gastroenterology, with 25-30 years of laparoscopic experience. |
| Non-simulation interventions and adjuncts* | Lectures on laparoscopic topics, laparoscopic techniques and learning outcomes of the box-trainers and VR-simulator were given prior to the simulation-training sessions on each course day. | | |

*Elements from Cheng et al. (2016)(24). Table 3 Key Elements to Report for Simulation-Based Research.
