# Supplementary material for: Stress responses in surgical trainees during simulation-based training courses in laparoscopy
Source: BMC Med Educ. 2024 Apr 12;24:407. doi: 10.1186/s12909-024-05393-3 (PMC11010405; doi:10.1186/s12909-024-05393-3)
Supplement: Supplementary file 4 — Supplementary Material 4 [file 12909_2024_5393_MOESM4_ESM.docx]

**Additional file 4:**

**The State-Trait Anxiety Inventory (STAI)**

**(modified version of Marteau & Bekker 1992)**

Read each statement and then circle the most appropriate number to the right of the statement to indicate how you feel right *now*, at this moment. There are no right or wrong answers. Do not spend too much time on any one statement but give the answer which seems to describe your present feelings best.

|  | Not at all | Somewhat | Moderately | Very much |
| --- | --- | --- | --- | --- |
| I feel calm | 1 | 2 | 3 | 4 |
| I am tense | 1 | 2 | 3 | 4 |
| I feel upset | 1 | 2 | 3 | 4 |
| I am relaxed | 1 | 2 | 3 | 4 |
| I feel content | 1 | 2 | 3 | 4 |
| I am worried | 1 | 2 | 3 | 4 |

Please make sure that you have answered all the questions.
